# Veteran suicide prevention learning collaborative: implementation strategy and processes

**DOI:** 10.3389/fpsyt.2024.1392218

**Published:** 2024-07-10

**Authors:** Joseph Mignogna, Patricia D. Russell, Elisa Borah, Craig J. Bryan, Lindsey L. Monteith, Kathryn Bongiovanni, Edgar Villareal, Claire A. Hoffmire, Alan L. Peterson, Jenna Heise, Nathaniel Mohatt, Sylvia Baack, Kimberly Weinberg, Marcy Polk, Meredith Mealer, Benjamin R. Kremer, James Gallanos, Alexis Blessing, Juliana Scheihing, Tabitha Alverio, Justin Benzer, Bryann B. DeBeer

**Affiliations:** ^1^ Rocky Mountain Mental Illness, Research, Education and Clinical Center (MIRECC) for Suicide Prevention, Rocky Mountain Regional Veterans Affairs (VA) Medical Center, Aurora, CO, United States; ^2^ Department of Physical Medicine and Rehabilitation, University of Colorado Anschutz Medical Campus, Aurora, CO, United States; ^3^ Steve Hicks School of Social Work, The University of Texas at Austin, Austin, TX, United States; ^4^ Dell Medical School, University of Texas at Austin, Austin, TX, United States; ^5^ Department of Psychiatry and Behavioral Health, Ohio State University, Columbus, OH, United States; ^6^ Veterans Affairs (VA) Center of Excellence for Suicide Prevention, Veterans Affairs (VA) Finger Lakes Health Care System, Canandaigua, NY, United States; ^7^ Department of Psychiatry, University of Colorado Anschutz Medical Campus, Aurora, CO, United States; ^8^ Veterans Affairs (VA) Veterans Integrated Service Network (VISN) 17 Clinical Resource Hub, Texas Valley Costal Bend Veterans Affairs (VA), Harlingen, TX, United States; ^9^ Office of Mental Health and Suicide Prevention, Veterans Affairs (VA) Central Office, Washington, DC, United States; ^10^ Department of Psychiatry and Behavioral Sciences, University of Texas Health Science Center at San Antonio, San Antonio, TX, United States; ^11^ Research and Development Service, South Texas Veterans Health Care System, San Antonio, TX, United States; ^12^ Department of Psychology, University of Texas at San Antonio, San Antonio, TX, United States; ^13^ Suicide Prevention Center of New York, Albany, NY, United States; ^14^ Zero Suicide Institute Faculty, Education Development Center, Waltham, MA, United States; ^15^ Division of Prevention and Community Research, Yale School of Medicine, New Haven, CT, United States; ^16^ Booz Allen Hamilton, Arlington, VA, United States; ^17^ Michael E. DeBakey Veterans Affairs (VA) Medical Center, Houston, TX, United States; ^18^ Central Texas Veterans Affairs (VA) Health Care System, Temple, TX, United States; ^19^ Veterans Affairs (VA) Portland Health Care System, Portland, OR, United States; ^20^ Veterans Affairs (VA) Eastern Colorado Health Care System, Aurora, CO, United States; ^21^ Veterans Integrated Service Network (VISN) 17 Center of Excellence for Research on Returning War Veterans, Waco, TX, United States; ^22^ Department of Psychiatry, Dell Medical School, University of Texas at Austin, Austin, TX, United States

**Keywords:** suicide prevention, veterans, learning collaborative, public health, community case study

## Abstract

The majority of Veterans who died by suicide in 2021 had not recently used Veterans Health Administration (VA) services. A public health approach to Veteran suicide prevention has been prioritized as part of the *VA National Strategy for Preventing Veteran Suicide*. Aligned with this approach, VA’s Patient Safety Center of Inquiry—Suicide Prevention Collaborative piloted a Veteran suicide prevention learning collaborative with both clinical and non-clinical community agencies that serve Veterans. The VA COmmunity LeArning CollaboraTive (CO-ACT) uses a quality improvement framework and facilitative process to support community organizational implementation of evidence-based and best practice suicide prevention strategies to achieve this goal. This paper details the structure of CO-ACT and processes by which it is implemented. This includes the CO-ACT toolkit, an organizational self-assessment, a summary of recommendations, creation of a blueprint for change, selection of suicide prevention program components, and an action plan to guide organizations in implementing suicide prevention practices. CO-ACT pilot outcomes are reported in a previous publication.

## Introduction

An average of 17.5 Veterans died by suicide daily in 2021 (1). Of these Veterans, 61.9% had not used Veterans Health Administration (VA) services in 2020 or 2021 (1). Thus, Veterans not using VA services are a critical subgroup to target in suicide prevention efforts.

VA has developed and implemented a multicomponent suicide prevention program that is unmatched by other healthcare system in the public or private sectors (2–5). In particular, VA has transitioned to implementing a public health approach to suicide prevention that calls on VA and non-VA clinical and community organizations to implement suicide prevention, intervention, and postvention services, as detailed in the VA’s *National Strategy for Preventing Veteran Suicide* (6).

The National Strategy (6) describes four pillars of suicide prevention: 1) Healthy and Empowered Veterans, Families, and Communities, 2) Clinical and Community Preventive Services, 3) Treatment, Recovery, and Support Services, and 4) Surveillance, Research, and Evaluation. VA implemented a new national initiative with these pillars in mind, namely, Community-Based Interventions for Suicide Prevention (CBI-SP). Through the CBI-SP initiative and embracing collaborations with community partnerships, VA has expanded its capacity to prevent suicide among Veterans not connected to VA care. The CBI-SP model encourages establishing community-based suicide prevention coalitions that target priority areas aimed at increasing: 1) identification of Veterans and family members within the community and increasing screening for suicide risk; 2) connectedness within the community and during improved care transitions; and 3) community-wide lethal means safety and safety planning.

Aligning with the CBI-SP initiative, the VA Patient Safety Center of Inquiry for Suicide Prevention (PSCI-SPC), a national suicide prevention research center sponsored by the VA National Center for Patient Safety, developed and tested the VA COmmunity LeArning CollaboraTive (CO-ACT). CO-ACT employs a learning collaborative model as one method to engage and partner with clinical and nonclinical community organizations to expand suicide prevention programming (7, 8). Prior literature details a collaborative learning model developed by the Institute for Healthcare Improvement, known as the Breakthrough Series (9). This model provides a structured and time-limited process (6- to 16-months) for healthcare organizations to learn from each other and subject matter experts. Participating teams then utilize quality improvement methods to translate that knowledge into action at their respective organizations. The Zero Suicide Institute also uses a learning collaborative method to assist organizations in implementing the Zero Suicide Model (10, 11). However, suicide prevention learning collaboratives beyond Zero Suicide are limited, and none target preventing suicide among military Veterans, a population with substantially higher suicide rates than non-Veteran adults. In 2021, after adjusting for sex and age, the rate of Veteran suicide deaths was 71.8% greater than non-Veteran adults (1).

The CO-ACT suicide prevention learning collaborative draws heavily from existing prevention models. Namely, the Mental Health Intervention Spectrum is rooted in the National Academy of Medicine’s (formerly named Institute of Medicine) prevention model and outlines strategies from promotion to recovery (12–14). While the Substance Abuse and Mental Health Services Administration (SAMHSA) prevention classification system was originally created to target substance misuse, it’s use is applicable to suicide prevention efforts (15). Using a Veteran suicide prevention lens, the learning collaborative focused on *Promotion* and *Prevention* strategies (12, 13, 16). *Promotion* efforts broadly target the general public. *Prevention* is divided into three stages that target increasingly specific populations through strategies that include: *universal prevention* (e.g., targets the general population, such as through suicide prevention awareness and education), *selective prevention* (e.g., targets individuals at increased risk for suicide), and *indicated prevention* (e.g., targets individuals at high risk for suicide due to a past suicide attempt or current suicidal ideation; 13).

CO-ACT utilizes the integrated—Promoting Action on Research Implementation in Health Services (i-PARIHS) framework (17). i-PARIHS framework identifies three components as key to successfully implement evidence-based and best practices: context, recipients, and the innovation. Considering these key components prior to initiating implementation can increase the likelihood of success. Context refers to the work setting in which implementation will occur and relates to multiple levels (i.e., local, organizational, external health system). An innovation that does not fit into a clinic’s ongoing workflow is unlikely to be successfully implemented. The recipient refers to the individuals impacted by implementation at the individual and collective team levels. Recipients must understand the implementation and be able to implement the innovation into practice. For example, if a selected implementation requires independently licensed providers, the recipients implementing it must be clinically licensed. The innovation is the specific practice or change the team aims to implement and should be clearly defined and provide an advantage over current practices. Receptivity to implement an innovation is impacted both by evidenced-based and practice-based knowledge. Harvey and Kitson (2015) described how the compatibility of a proposed change can be enhanced by “aligning external explicit evidence [for an innovation] with local priorities and practice…” (p. 4) (17). Attending to these three key components can facilitate rapid implementation by preventing stuck points and improving the likelihood of implementation success.

CO-ACT has four aims: 1) create sustained organizational change so that organizations can adequately respond to Veteran suicide risk; 2) provide technical assistance, support, and education in building a suicide prevention program within community organizations; 3) build relationships and collaborations between the VA and community organizations to support suicide prevention; and 4) build relationships between community organizations to strengthen a Veterans suicide prevention safety net in the community. From 2020–2021, CO-ACT was pilot tested with clinical and non-clinical organizations across the broader Denver and Colorado Springs, Colorado region. The aim of the current paper is to detail CO-ACT’s structure and processes. In doing so, the authors seek to increase knowledge of mechanisms of learning collaboratives for Veteran suicide prevention, specifically methods that facilitate rapid implementation of needed Veteran suicide prevention strategies into community organizations.

## Context for an initial test of a suicide prevention learning collaborative

**Figure 1:**
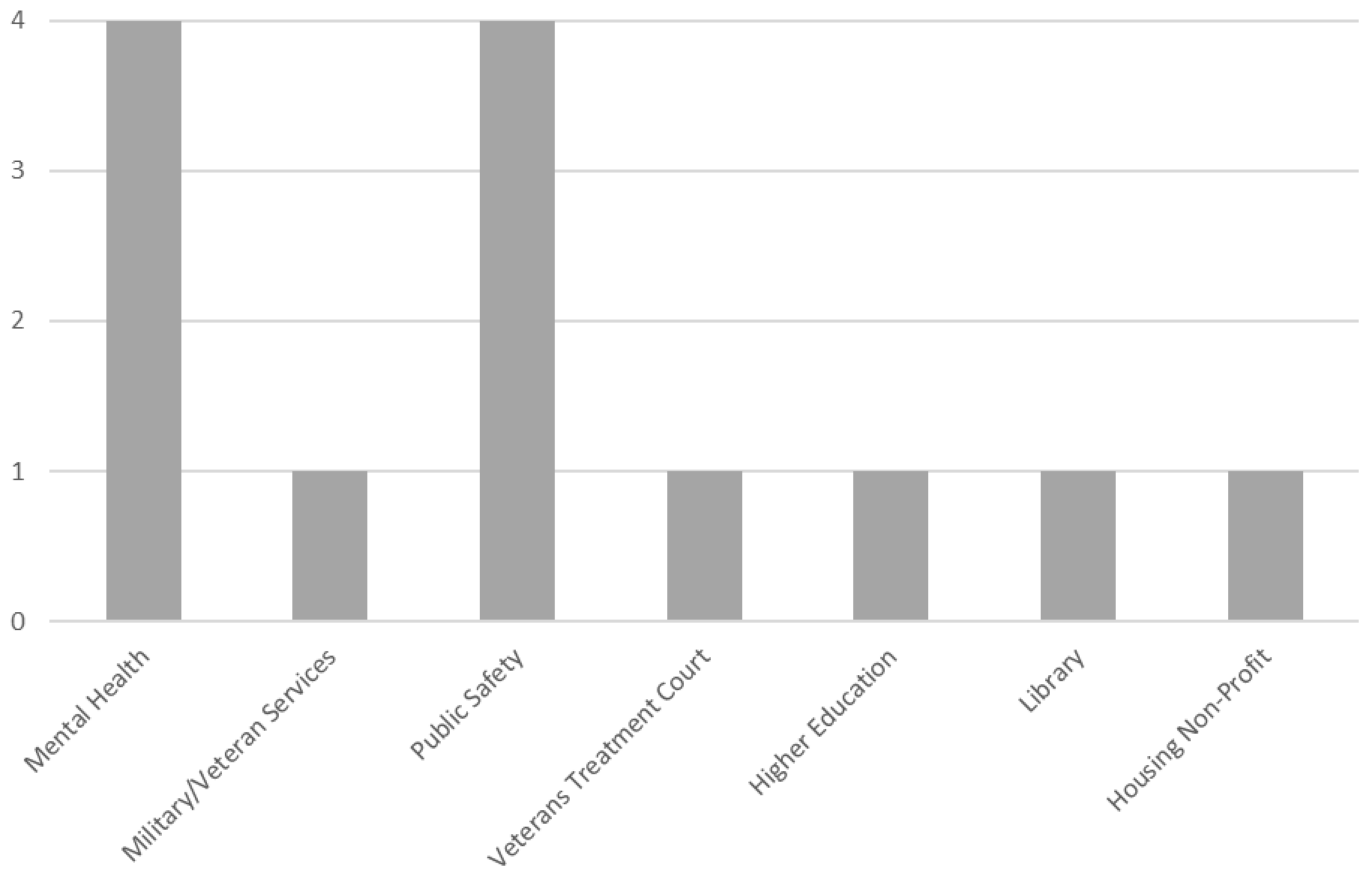
Types of organizations in CO-ACT learning collaborative pilot (8).

Colorado has notably high rates of suicide. In 2021, Veterans in Colorado had a significantly higher rate of suicide compared to the national Veteran suicide rate and the national general population suicide rate (1). To expand and improve upon existing suicide prevention efforts across the state in its pilot test, CO-ACT employed community mapping to understand the landscape of community organizations serving Veterans in the greater Denver area and support organization recruitment. Further, snowball recruitment was used to identify and invite other clinical and non-clinical organizations serving Veterans to participate. Local VA and community leaders provided recommendations and/or connected the CO-ACT team to these potential organizations. In 2020, 13 clinical and non-clinical community organizations were invited and participated in a pilot test of CO-ACT. Five of these organizations provided mental health services to Veterans and/or Military Service Members, while the remaining eight provided non-healthcare services [see **Figure 1** for additional details, as reported in DeBeer et al., 2023b (8)]. At the discretion of each organization, each organization’s team size ranged from one to four members.

## Essential program components of CO-ACT: suicide prevention learning collaborative

CO-ACT was adapted for delivery using an entirely virtual learning collaborative (i.e., videoconference calls via Zoom™ and Microsoft Teams™) following early COVID-19 pandemic in-person meeting restrictions. CO-ACT occurred over 16 months, and consisted of 6 quarterly collaborative group meetings and 15 monthly individual organizational team facilitation calls. Implementation activities were guided by the Institute for Healthcare Improvement (IHI) model (9), existing prevention models, and suicide prevention programs/frameworks (6, 11, 18–24). The iPARIHS framework (17), in conjunction with Plan-Do-Study-Act (PDSA) cycles (25), guided implementation and quality improvement planning and action.

## CO-ACT toolkit and program components

Program Components (i.e., the specific suicide prevention programming practices teams select to implement at their respective organizations) included suicide prevention, intervention, and postvention service options identified in one or more of four existing and prominent Veteran, active military, or civilian suicide prevention suicide prevention programs or models. Namely, these programs/models comprised of the Department of Veterans Affairs Suicide Prevention Program (6, 18), the Defense Suicide Prevention Program (DoD) (20, 21), the Zero Suicide program (11, 22), and the Division of Violence Protection National Center for Injury Prevention and Control-Centers for Disease Control (CDC) and Prevention program (23, 24). These four suicide prevention programs/models were compared to identify all possible suicide prevention strategies beneficial to VA and community suicide prevention programming (2). Once all distinct program components were identified, similar strategies were combined and re-defined to ensure integrity of the original definition.

This resulted in a comprehensive list of the suicide prevention program components detailed in the CO-ACT toolkit. Each program component was categorized according to two existing prevention frameworks. Specifically, the Mental Health Intervention Spectrum (adapted from the National Academy of Medicine Continuum of Care Model (12–14, 16) and the SAMHSA Prevention Model (15). The intent with labeling each program component according to these frameworks was to assist organizations in understanding the spread of their program components over different types of prevention. Consistent with Reason’s Swiss Cheese Model, prevention practices are strongest when they are spread over multiple types (26, 27).

Program components were also categorized as either a *best practice* or an *evidence-based practice* (28–33). *Best practices* are generally accepted treatments, techniques, or methods by health care experts that are used by professionals as appropriate treatments for certain disorders that have proven helpful over time and that are conferred as being measurable, replicable, and notably successful (31, 33), In contrast, an *evidence-based practice* is one that has undergone scientific evaluation to validate its effectiveness and which is recognized in peer-reviewed scientific journals (28–33).

Each program component in the CO-ACT toolkit was labeled according to the Zero Suicide classification to assist teams in determining how practices implemented through CO-ACT fit with the Zero Suicide framework (11). Zero Suicide categorizes suicide prevention components as: *Lead* (i.e., interventions promoting suicide prevention at a systems level), *Train* (i.e., training staff to promote high quality care services), *Identify* (i.e., identifying those at risk using a screening tool), *Engage* (i.e., using suicide care management plans for those at risk), *Treat* (i.e., using evidenced-based treatments and other best practices to reduce risk), *Transition* (i.e., using best practices for care coordination to ensure the Veteran remains connected to services), and *Improve* (i.e., using quality improvement to continually improve suicide prevention practices) (11).

## Procedures

### Pre-collaborative assessment

After agreeing to participate in CO-ACT, each member organization completed an organizational self-study and interview. Specifically, each organization completed a modified Zero Suicide survey focused on identifying each organizational structure and existing Veteran suicide prevention practices (11). These data were used to inform a follow-up hour-long individual interview aimed at providing the VA CO-ACT facilitation leads with a fuller picture of each organization’s existing structure and suicide prevention program components. Together, this information was used to create a summary of specific Veteran suicide prevention component recommendations that each member organization could consider implementing in the near future.

### Learning collaborative structure

Following a 4-hour kick-off group meeting (see **Table 1** for content of group quarterly meetings), each team participated in their first monthly 1-hour coaching call with VA CO-ACT facilitation leads. In this call, VA CO-ACT facilitation leads engaged each team in a collaborative review of information collected during the organizational self-study and interview. VA CO-ACT facilitation leads identified and prioritized potential suicide prevention program components for implementing over the course of the learning collaborative. Factors impacting selection and prioritization of program components included consideration of each organization’s current suicide prevention programming, along with its leadership interest and available resources. Priority was placed on implementing sustainable programming (e.g., having leadership approve a new training policy on suicide risk assessment practices regarding who is trained, how, and when before an organization initiates efforts to train staff), as well as a desire to have as large of an impact as possible on reducing Veteran suicide. This information was used to develop each team’s Blueprint (i.e., a roadmap used to guide selection of the suicide prevention program components of interest to each organization) and Action Plan (i.e., detailed plan created to enhance the likelihood of implementation success; see **Tables 2**, **3**).

**Table 1:** Illustrative example of Co-ACT quarterly meeting presentations.

Quarterly Meeting	Presentation
1	Introduction to the Learning Collaborative and Processes for Creating a Blueprint
2	Processes for Creating an Action Plan
3	VA Organizational Navigation and Resources
4	Suicide Prevention Programming Training Resources
5	VA Mental Health Navigation and Resources
6	Wrap up of the Collaborative and Next Steps

**Table 2:** Blueprint and definitions: clinical example.

BLUEPRINT COMPONENTS									
Change Target	Aim Statement	Short-term Goal	Long-term Goal	Program Component	Prevention Strategies: P/UP/SP/IP ^+^	SAMHSA^++^ Prevention Types	Best Practice vs. Evidence-Based	Outcomes	Metrics
DEFINITIONS									
A change an organization makes to achieve a desired outcome	What an organization aims to achieve; include outcome(s)	Implementations reasonable to achieve on a short timeline	The outcome an organization aims to achieve	An organization’s primary components of its suicide prevention program	Suicide prevention program components specific to target populations	Provides information regarding the spread of program components	Using both evidence-based and best practices lead to a diversified program targeting multiple levels of prevention	Measurable performance goal that demonstrates success of the change	The specific data collected to measure or determine progress
CLINICAL EXAMPLE									
Improve screening rates of Veteran suicide risk	Create a standardized process for suicide risk screening, including an SOP^+++^	- Draft SOP - Select screening measures	- Finalize SOP and screening procedures	Integrate suicide prevention into organizational policy	Universal Prevention	Environmental Strategies	Best Practice	Finalized SOP is implemented	Finalized SOP is implemented
Improve screening rates of Veteran suicide risk	Train all agency staff on new suicide prevention SOP	Conduct in-person training for 75% of the staff and record virtual training for remaining staff	All staff trained in suicide prevention procedures	Suicide prevention training for staff	Universal Prevention	Prevention Education	Best Practice	100% of staff trained	- Staff knowledge of SOP post-training survey Number of staff trained • Number of clients identified as at risk
Improve screening rates of Veteran suicide risk	Screen incoming Veterans for suicide risk	Screen 25% of Veterans who interact with the organization within the next 3-months	Screen 100% of Veterans who interact with the organization	Screening	Universal Prevention	Environmental Strategies	Evidence-based	Number of Veterans screened in 3 months	Number of Veterans screened

^+^P/UP/SP/IP, Prevention Strategies: Promotion, Universal Prevention, Selective Prevention, Indicated Prevention; ^++^SAMHSA, Substance Abuse and Mental Health Service Administration; ^+++^SOP, Standard Operating Procedure.

**Table 3:** Action plan and definitions: non-clinical example.

ACTION PLAN COMPONENTS											
Change Target/Aim	Preparatory Tasks	Plan	Do	Study	Act	Implementation Considerations			Action Items	Responsible Person(s)	Timeline
						Context	Innovation	Recipient			
DEFINITIONS											
Change an organization makes to achieve a desired outcome	Tasks that are necessary to complete prior to starting the implementation	Create a plan to test the implementation	Perform a small test of the implementation	Analyze and study the results of the data	Plan for next steps based on lessons learned from previous PDSA cycle	The setting in which the implementation takes place	The change you are working on implementing	Individuals/teams who are affected by or will influence the implementation efforts	Identify tasks or resources needed to carry out the implementation	Identify person responsible to implement each action item or change	Identify the timeline needed to implement each action item or change
NON-CLINICAL EXAMPLE											
Improve access to care and outcomes for Veterans at-risk for suicide Develop an SOP+ for when and how to refer an at-risk Veteran	• Obtain example SOPs from other non-clinical orgs • Identify local referral options • Contact referral options to establish relationships and referral procedures	Create list of potential SOP components and consult organizational leadership to solidify	Draft SOP for when and how to refer Veterans identified as at-risk for suicide	Request and review feedback from organizational leadership and VA team	Revise SOP as needed	Clarify use of SOP within flow of other organizational tasks	Align SOP within organizational needs	Create user-friendly SOP for all staff, regardless of role within organization	Obtain example SOPs	Theresa	02/2020
									Identify and contact local referral options	Joe & Allison	03/2020
									Meet with leadership and introduce SOP options	Angelina	05/2020
									Draft SOP	All team	06/2020
									Finalize SOP	Theresa	08/2020
Improve access to care and outcomes for Veterans at-risk for suicide Train all staff on new SOP	• Set dates for staff to attend or complete recorded training • Create recording of training for those who cannot attend in-person	Determine appropriate level of staff training needed (i.e., foundational, advanced, or expert)	Conduct in-person training and share recorded training	Address questions that arise during training	Arrange for additional trainings if needed	Consider frequency of training	Training reflects the non-clinical nature of the organization	Training is within scope of practice for employees	Set training dates	Kristin	07/2020
									Create presentation	Theresa & Melissa	08/2020
									Revise proposed training content	All team	09/2020
									Conduct and record in-person training	Ira & Jax	10/2020

Each team’s evolving Blueprint and associated Action plan were reviewed during monthly facilitation calls with the CO-ACT learning collaborative leader. Additionally, each organization provided brief updates about these materials during the second through the sixth quarterly group learning collaborative meetings. Quarterly group meetings were also used to educate members about numerous areas associated with Veteran care and suicide prevention. Topics covered can vary between CO-ACT cohorts, as the learning collaborative leader is encouraged to add or remove topics to accommodate specific requests. The topics discussed during the initial pilot at quarterly group meetings are detailed in **Table 1**.

Following selection of program components to implement, each CO-ACT team considered how implementation success could be impacted by the *context*, *innovation*, and *recipient*, as defined by iPARIHS model (17). PDSA cycles provided a model to guide a continuous approach to quality improvement efforts (25) that allowed for ongoing revisions to each PDSA cycle, until a practice is well-integrated into an organization’s operations. This quality improvement approach facilitates a process to assist teams in breaking down larger implementations into manageable tasks.

### CO-ACT quality improvement process

**Figure 2:**
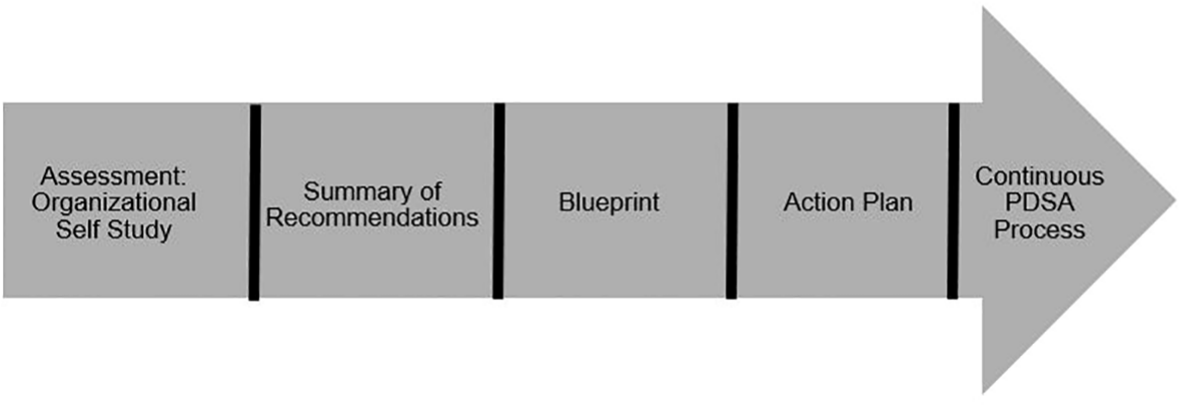
CO-ACT quality improvement process.

**Figure 2** depicts the CO-ACT 5-step process to facilitate rapid implementation of Veteran suicide prevention in partner organizations. Each step in this process is described in detail below.

#### Step 1: assessment: organizational self-study and interview

As previously mentioned, all teams were required to complete an organizational self-study survey and an interview prior to the start of CO-ACT. The organizational self-study was modified from Zero Suicide to be specific to Veteran suicide prevention (11, 22). The survey consists of questions regarding the functions of the organizations and populations served. Additionally, it assesses information regarding each organization’s current Veteran suicide prevention programming and goals for participation in CO-ACT. The interview following the survey provided an opportunity for the VA CO-ACT facilitation leads to review the organization’s current programming, relationship with the VA, and relationship with other collaborative organizations.

#### Step 2: summary of recommendations

After completing the organizational self-study and the interview, CO-ACT leads reviewed the results and organized them into a summary of recommendations. The learning collaborative leader sought to understand the population served by the organization, paying particular attention to how much of that population is comprised of Veterans. Some organizations were Veteran-focused, serving only Veterans, whereas others served much smaller concentrations. This information influenced how implementation was approached at each organization. The VA CO-ACT facilitation leads reviewed practices and identified gaps in the organization’s Veteran suicide prevention programming, then collaboratively worked with each organizational team to determine the top three areas of Veteran suicide prevention to begin implementing best practices and/or evidence-based practices.

#### Step 3: Blueprint

Each organization’s team built a Blueprint for change (see **Table 2** for an illustrative Blueprint of an organization that delivers mental health services) that is subsequently enacted using an action plan. A Blueprint provides a roadmap to guide *what* suicide prevention programming components an organization implements, whereas the Action Plan details *how* to enact the Blueprint, similar to a project management plan. A Blueprint consists of a change target, aim statement, necessary tasks in preparation for implementing suicide prevention programming, short- and long-term goals, outcomes, and metrics. A change target, which refers to the change that the team makes to improve the desired outcome at their organization, may have one or multiple sub-component implementations connected to it (9). The aim statement is a succinct and measurable statement about what the organization aims to accomplish and for which specific population, and identifies the outcomes to be assessed. Teams were encouraged to regularly revisit their aim statement to prevent project drift, and as needed, to revise or refocus this statement while implementing changes. Both short- and long-term goals were identified and defined to reflect actions with a specified target completion date (9). Teams were encouraged to identify goals believed to be most effective in producing intended results (i.e., see “Program Component Selection” section below). Short-term goals can typically be completed in 1 to 3 months, while long-term goals reflect an end state the organization aims to accomplish, usually in 12 to 16 months.

##### Program component selection

The VA CO-ACT facilitation leads assisted each team in selecting program components that best fit each organization. Consistent with the VA’s community-based suicide prevention priorities (6), each Co-ACT team was encouraged to consider the top three aforementioned Veteran suicide prevention program components: 1) identifying service members, Veterans, and their families and screening for suicide risk, 2) promoting connectedness and improving care transitions, and 3) increasing lethal means safety and safety planning. Teams were asked to consider both the level of involvement their respective organizations have with at-risk Veterans and were asked questions about feasibility (e.g., access to necessary resources) to assist in selecting strategies best suited for their organization. For example, while mental health organizations may find evidence-based treatments and tools to assess for suicide risk a priority for their practice, organizational leaders providing services to Veterans outside of health care may find strategies such as providing promotional materials and training staff in military cultural competency the best fit.

Teams were asked to define and track intended outcomes of newly implemented suicide prevention practices. While the ultimate outcome was to reduce Veteran suicide deaths, measuring this outcome as a result of implementing any one suicide prevention practice is difficult due to the rarity of the event within a single organization. Consequently, other implementation-related outcomes (e.g., number of patients assessed for Veteran status, number of providers trained in suicide prevention and lethal means safety) provided means for tracking implementation metrics and suicide prevention outcomes.

#### Step 4: action plan

After creating the Blueprint, each team worked to build the Action Plan. The Action Plan provided a project management plan to focus organizational efforts in rapid implementation of the identified change target (see **Table 3**). The Action Plan starts with the same change target and aim identified in the Blueprint, then a PDSA cycle is used to plan subsequent implementation activities (25). A critical piece of the PDSA cycle is determining any preparatory-PDSA cycle tasks. For example, if a training is planned as one cycle, the organization needs to first determine the type of training they want to provide. In the PDSA cycle, the first step is the *Plan*. The team and learning collaborative leader worked together to create a plan to test the implementation (25). The second step is *Do*. In this step, a test of the implementation is conducted. In the third step, *Study*, results of the test are analyzed. In the final step, *Act*, lessons learned in implementation are used to plan for subsequent cycles. Oftentimes, each PDSA cycle results in further clarity regarding what should be done next. Clarity surrounding who is moving the work of the implementation forward and the timeline is key; when this is not clear, breakdowns in implementation can occur. The Action Plan provides space to determine who is responsible for each step during implementation, and completion dates are concurrently determined. Although timelines can vary, each action item is typically due within a month, corresponding to the next scheduled facilitation call.

#### Step 5: continuous PDSA process

Once organizations move through a PDSA cycle and successfully implement new suicide prevention programing, the organization will return to the Blueprint to determine which program component to implement next. Often, engaging in a PDSA cycle process brings to light additional program components to implement, which is encouraged in the spirit of creating an environment of continuous quality improvement. A matrix is used to map the diversity of an organization’s programming (see illustrative Matrix in **Table 4**). Once existing suicide preventing programing is mapped using this matrix, an organization can examine diversification of their programming to assist selection of what to implement next. For example, if an organization is using all universal prevention strategies that are community-based processes, the organization could consider implementing a different classification strategy, in order to promote a diversity of prevention strategies. This process reveals which types of prevention in each organization’s suicide prevention programming overlap, and which are different. Optimally, each organization implements a wide range of prevention strategies. However, organizations that do not provide clinical services will likely have fewer selective prevention and indicated prevention strategies.

**Table 4:** Illustrative example of an organization’s suicide prevention program component matrix.

		Mental Health Intervention Spectrum Prevention Types			
		Promotion	Universal Prevention	Selective Prevention	Indicated Prevention
		^1^Military Cultural Competency Training			
SAMHSA Prevention Strategies	Information Dissemination		Promotional Materials		
	Prevention Education		Suicide prevention training for staff		Veteran suicide prevention clinical consultation
	Positive Alternatives			Post-suicide support for survivors	
	Environmental Strategies		Screening for Veteran status; Quality Improvement		Investigate every suicide/Root cause analysis
	Community-based Processes		Collaborative Partnerships		
	Identification of Problems and Referral to Services				Flagging for Veteran status in the medical record

^1^Promotion strategies do not have a corresponding SAMHSA prevention strategy.

## Discussion

The current paper described the structure and processes of CO-ACT, a Veteran suicide prevention focused learning collaborative (7) that employs a quality improvement framework to enact a public health approach toward reducing suicide. CO-ACT uses a learning collaborative model (9) to implement best and evidence-based suicide prevention program practices applicable to Veterans (6, 11, 18–24) in community organizations providing clinical or non-clinical services to Veterans. By utilizing a continuous quality improvement process (i.e., PDSA cycles) (25) and the i-PARIHS model (17) to guide implementation efforts, this learning collaborative was designed to enact sustainable Veteran suicide prevention programming into community organizations (8). With this intention, priority was given to implementing programming that was likely to be sustained after the learning collaborative ended. For example, before implementing a training for staff in suicide risk assessment, a new training policy was first implemented on who should be trained, when, and how often staff should be retrained.

The format and structure of the learning collaborative allowed teams to have a framework from which to: 1) conceptualize the current state of their program; 2) determine how to move forward in building an internal Veteran suicide prevention program; and to 3) utilize existing internal and external resources to support a continuous quality improvement process. As reported in DeBeer and colleagues (2023), organizational teams found these processes to be acceptable and feasible, and facilitated significant implementation of suicide prevention programming across all partnering organizations (8). Further, as reported in DeBeer and Colleagues (2024), participating in CO-ACT resulted in an increase in the quantity and quality of relationships/partnerships between participating community organizations and the VA (34).

In contrast to how learning collaboratives typically focus on healthcare systems (9), the current collaborative included non-healthcare organizations. This was done to be consistent with the VA’s public health approach to suicide prevention (6), reaching Veterans at opportunities outside of healthcare interactions. Decisions were also made during the course of this pilot to consolidate program materials as a means of improving their usability. Specifically, the initial CO-ACT toolkit was conceptualized as a toolkit with a companion workbook. However, informal feedback from organizational teams and co-investigators suggested these materials should be package into one document. Consequently, the workbook was integrated into the toolkit to provide a single resource for partner organizations to utilize during CO-ACT.

VA CO-ACT facilitation leads collaboratively worked with each organization’s team in an effort to overcome challenges to implementing recommended suicide prevention programming. There were two commonly encountered barriers. First, organizations often lacked necessary resources (e.g., staffing shortages) and/or had staffing with significant constraints on their time. Second, not all organizations had data platforms available to easily track the implementation of suicide prevention programming, and tracking is an essential component to engaging in a continuous quality improvement process.

In light of encouraging findings from this pilot (8), subsequent tests are applying the same structure and processes of CO-ACT to a learning collaborative focused only on VA Community Care organizations (i.e., health care organizations the VA contracts with to provide Veteran health care services). Learning collaboratives can serve as a powerful vehicle for enhancing suicide prevention practices across organizations and offer promise as a solution to address Veteran suicide deaths in community settings.

## Data availability statement

The original contributions presented in the study are included in the article. Further inquiries can be directed to the corresponding author.

## Author contributions

JM: Conceptualization, Writing – original draft, Writing – review & editing. PR: Conceptualization, Writing – original draft, Writing – review & editing. EB: Writing – review & editing. CB: Writing – review & editing. LM: Writing – review & editing. KB: Writing – review & editing. EV: Writing – review & editing. CH: Writing – review & editing. AP: Writing – review & editing. JH: Writing – review & editing. NM: Writing – review & editing. SB: Writing – review & editing. KW: Writing – review & editing. MP: Writing – review & editing. MM: Writing – review & editing. BK: Writing – review & editing. JG: Writing – review & editing. AB: Writing – review & editing. JS: Writing – review & editing. TA: Writing – review & editing. JB: Conceptualization, Writing – review & editing. BD: Conceptualization, Writing – original draft, Writing – review & editing.
